# [^99m^Tc]Tc-DB15 in GRPR-Targeted Tumor Imaging with SPECT: From Preclinical Evaluation to the First Clinical Outcomes

**DOI:** 10.3390/cancers13205093

**Published:** 2021-10-12

**Authors:** Berthold A. Nock, Aikaterini Kaloudi, Panagiotis Kanellopoulos, Barbara Janota, Barbara Bromińska, Dariusz Iżycki, Renata Mikołajczak, Rafał Czepczynski, Theodosia Maina

**Affiliations:** 1 Molecular Radiopharmacy, INRaSTES, NCSR “Demokritos”, 15310 Athens, Greece; nock_berthold.a@hotmail.com (B.A.N.); katerinakaloudi@yahoo.gr (A.K.); kanelospan@gmail.com (P.K.); 2 National Centre for Nuclear Research, Radioisotope Centre POLATOM, 05-400 Otwock-Świerk, Poland; barbara.janota@polatom.pl (B.J.); renata.mikolajczak@polatom.pl (R.M.); 3 Department of Endocrinology, Metabolism and Internal Diseases, Poznan University of Medical Sciences, 60-355 Poznań, Poland; barbarabrominska@ump.edu.pl (B.B.); czepczynski@ump.edu.pl (R.C.); 4 Department of Cancer Immunology, Poznan University of Medical Sciences, 61-866 Poznań, Poland; dmizy@ump.edu.pl

**Keywords:** gastrin-releasing peptide receptor-antagonist, tumor-targeting, diagnostic imaging, breast cancer diagnosis, prostate cancer diagnosis, technetium-99m radiotracer

## Abstract

**Simple Summary:**

Radiolabeled gastrin-releasing peptide receptor (GRPR)-antagonists have been proposed for diagnostic imaging and radionuclide therapy—theranostics—of human tumors, including prostate (PC) and breast cancer (BC). We herein present [^99m^Tc]Tc-DB15, a SPECT radiotracer based on the potent GRPR-antagonist [_D_Phe^6^,LeuNHEt^13^]BBN(6-13). After Sar^11^/Gly^11^-replacement an acyclic tetraamine was coupled at the N-terminus via a spacer allowing for stable binding of the diagnostic radiometal Tc-99m. The forming [^99m^Tc]Tc-DB15 radiotracer displayed high in vitro uptake in GRPR-expressing mammary (T-47D) and prostate cancer (PC-3) cells. Furthermore, it showed an attractive biodistribution profile in mice bearing T-47D or PC-3 xenografts. A pilot proof-of-principle study of [^99m^Tc]Tc-DB15 in PC and BC patients applying SPECT is currently under way. Promising results from the first two BC patients are presented herein.

**Abstract:**

Diagnostic imaging and radionuclide therapy of prostate (PC) and breast cancer (BC) using radiolabeled gastrin-releasing peptide receptor (GRPR)-antagonists represents a promising approach. We herein propose the GRPR-antagonist based radiotracer [^99m^Tc]Tc-DB15 ([^99m^Tc]Tc-N_4_-AMA-DGA-_D_Phe^6^,Sar^11^,LeuNHEt^13^]BBN(6-13); N_4_: 6-carboxy-1,4,8,11-tetraazaundecane, AMA: aminomethyl-aniline, DGA: diglycolic acid) as a new diagnostic tool for GRPR-positive tumors applying SPECT/CT. The uptake of [^99m^Tc]Tc-DB15 was tested in vitro in mammary (T-47D) and prostate cancer (PC-3) cells and in vivo in T-47D or PC-3 xenograft-bearing mice as well as in BC patients. DB15 showed high GRPR-affinity (IC_50_ = 0.37 ± 0.03 nM) and [^99m^Tc]Tc-DB15 strongly bound to the cell-membrane of T-47D and PC-3 cells, according to a radiolabeled antagonist profile. In mice, the radiotracer showed high and prolonged GRPR-specific uptake in PC-3 (e.g., 25.56 ± 2.78 %IA/g vs. 0.72 ± 0.12 %IA/g in block; 4 h pi) and T-47D (e.g., 15.82 ± 3.20 %IA/g vs. 3.82 ± 0.30 %IA/g in block; 4 h pi) tumors, while rapidly clearing from background. In patients with advanced BC, the tracer could reveal several bone and soft tissue metastases on SPECT/CT. The attractive pharmacokinetic profile of [^99m^Tc]DB15 in mice and its capability to target GRPR-positive BC lesions in patients highlight its prospects for a broader clinical use, an option currently being explored by ongoing clinical studies.

## 1. Introduction

The expression of gastrin releasing peptide receptors (GRPRs) in a series of human tumors has provided the rationale for the application of anti-GRPR peptide radioligands in cancer diagnosis and therapy following a patient-tailored theranostic approach [1,2,3]. High levels of GRPR-expression have been indeed documented in excised patient biopsy specimens from prostate cancer (PC), especially in its early stages [4,5,6,7,8], breast cancer [9,10,11], gastrointestinal stroma tumors [12] and other human cancers [13,14]. The design of safe and effective radionuclide carriers to pathological GRPR-positive lesions was initially based on the amphibian tetradecapeptide bombesin (BBN, Pyr-Gln-Arg-Leu-Gly-Asn-Gln-Trp-Ala-Val-Gly-His-Leu-Met-NH_2_) and its octa/nonapeptide C-terminal fragments [1,2]. The resulting radioligands behaving as typical GRPR-agonists bound to the GRPR and rapidly internalized in cancer cells after intravenous injection (iv). At the same time, they activated the GRPR, eliciting a range of adverse effects mainly in the gastrointestinal system [15,16,17]. For example, such potent side effects were made evident during systemic radiotherapy of hormone refractory PC using [^177^Lu]Lu-AMBA ([^177^Lu]Lu-DOTA-Gly-*p*-aminomethylaniline-Gln-Trp-Ala-Val-Gly-His-Leu-Met-NH_2_) in a pilot study involving a small number of patients [18,19]. 

Soon thereafter, a shift of paradigm to GRPR-antagonists occurred [3,20] with a wide range of radiolabeled GRPR-antagonists (or GRPR-radioantagonists from now on) being developed and tested via systematic preclinical structure-activity relationships studies (SARs). This transition in nuclear medicine was facilitated by numerous existing GRPR-antagonist motifs, developed in previous years either as “cold” (non-radioactive) anticancer drugs, or as molecular tools for elucidating the pharmacology of the bombesin receptor family [3,21,22,23,24]. As a rule, GRPR-antagonists were generated by structural interventions on the C-terminal BBN(6/7-14) fragment, and in particular on the end Leu-Met-NH_2_-dipeptide [3,21]. As expected, GRPR-antagonists turned out to be safer for human use in view of their inability to activate the GRPR. Although this feature went hand-in-hand with their lack of internalization in cancer cells, GRPR-radioantagonists did achieve significant uptake and retention in tumor lesions in mice and in patients. In addition, they cleared more rapidly from background tissues, even from GRPR-rich organs, such as the pancreas, compared with their agonist-based counterparts, eventually resulting in superior pharmacokinetic profiles [3]. A higher metabolic stability in the blood stream turned out to be another advantageous feature of GRPR-radioantagonists [25,26,27].

During our search for clinically useful GRPR-radioantagonists, we have often employed the [_D_Phe^6^,LeuNHEt^13^]BBN(6-13) motif [27,28,29]. This potent GRPR-antagonist resulted after truncation of Met^14^ and ethylamidation of Leu^13^ in the [_D_Phe^6^]BBN(6-14) fragment [30,31]. Coupling of suitable chelators at the N-terminus via different linkers gave rise to a series of analogs, amenable to radiolabeling with clinically appealing radiometals. As a result, single photon emission computed tomography (SPECT; Tc-99m, In-111) or positron emission tomography (PET; Ga-68) diagnostic imaging and radionuclide therapy (Lu-177) could be performed [7,25,26,29,32]. Specifically, for labeling with the preeminent SPECT radiometal Tc-99m we have employed an acyclic tetraamine chelator. The tetraamine chain positioned at the equatorial plane of an octahedron is wrapped around the *trans*-[^99m^Tc]Tc(O)_2_^+^ core forming stable monocationic complexes [33]. The first such radiotracer, [^99m^Tc]Tc-DB1 (DB1, demobesin 1, [N_4_′-DGA-_D_Phe^6^,LeuNHEt^13^]BBN(6-13); N_4_′, 6-{*p*-[(carboxymethoxy)-acetyl]-aminobenzyl}-1,4,8,11-tetraazaundecane); DGA, diglycolic acid), showed attractive in vivo profile in mice bearing human PC-3 xenografts [20,34]. Further development of a series of [^99m^Tc]Tc-DB1 mimics followed by structural interventions on the spacer or/and the peptide chain [35,36]. 

**Figure 1:**
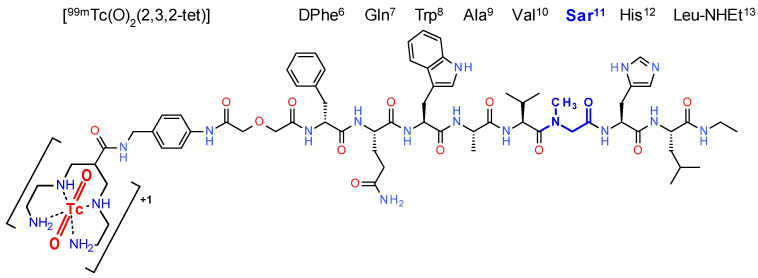
Chemical structure of [^99m^Tc]Tc-DB15 showing the *trans*-[^99m^Tc]Tc(O)_2_^+^-core incorporated in the acyclic tetraamine chain under formation of a monocationic octahedral radiometal-chelate; Sar^11^ replacing Gly^11^ in the parent peptide sequence is highlighted in blue and bold.

In the present study, we decided to explore the effect of Gly^11^ by Sar^11^ (sarcosine, N-methylglycine) substitution on the profile of a new [^99m^Tc]Tc-DB1 mimic, [^99m^Tc]Tc-DB15 ([^99m^Tc]Tc-N_4_-AMA-DGA-_D_Phe^6^,Sar^11^,LeuNHEt^13^]BBN(6-13); N_4_: 6-carboxy-1,4,8,11-tetraazaundecane, AMA: *p*-aminomethyl(aniline); Figure 1). Previous studies have shown that replacement of Gly^11^ by DAla^11^ resulted in radioligands of higher metabolic stability, but without notable improvement of end-pharmacokinetics [35,37]. Studying the profile of [^99m^Tc]Tc-DB15 in mice, we could confirm a high metabolic stability in the blood stream. We could also observe specific radioligand uptake in two GRPR-positive tumor animal models, namely, in human prostate PC-3 and breast T-47D cancer xenografts in immunosuppressed mice combined with a rapid background clearance. In a first pilot translational study, SPECT/CT diagnostic imaging was performed in two advanced BC patients, confirming the promising attributes of [^99m^Tc]Tc-DB15 for use in nuclear oncology. 

## 2. Materials and Methods

### 2.1. Peptides, Inhibitors and Radionuclides 

DB15 (N_4_-AMA-DGA-_D_Phe^6^,Sar^11^,LeuNHEt^13^]BBN(6-13); N_4_: 6-carboxy-1,4,8,11-tetraazaundecane, AMA: aminomethyl-aniline, DGA: diglycolic acid; Figure 1) was provided by PiChem (Raaba-Grambach, Austria; https://www.pichem.at/ (accessed on 7 October 2021)). [Tyr^4^]BBN (Tyr^4^-bombesin, Pyr-Gln-Arg-Tyr-Gly-Asn-Gln-Trp-Ala-Val-Gly-His-Leu-Met-NH_2_) was purchased from Bachem AG (Bubendorf, Switzerland). The NEP-inhibitor phosphoramidon (PA; phosphoramidon disodium dehydrate, N-(α-rhamnopyranosyloxyhydroxyphosphinyl)-L-leucyl-L-tryptophan × 2Na × 2H_2_O) was obtained from PeptaNova GmbH (Sandhausen, Germany). Technetium-99m in the form of [^99m^Tc]NaTcO_4_ was collected by elution of a [^99^Mo]Mo/[^99m^Tc]Tc-generator (Ultra-Technekow^TM^ V4 Generator, Curium Pharma, Petten, The Netherlands). A solution of [^125^I]NaI in 10^−5^ M NaOH (10 µL) was purchased from Perkin Elmer (Waltham, MA, USA). 

### 2.2. Radiolabeling—Radioanalytical Control 

For the preclinical studies, labeling of DB15 with Tc-99m was performed in alkaline pH and room temperature using SnCl_2_ as a reductant and citrate as a transfer ligand, following a published protocol [35]. Labeling yields and radiochemical purities were monitored by a combination of instant thin layer chromatography (ITLC) and high performance liquid chromatography (HPLC) adopting twin photometric and radiometric detection modes (HPLC equipment is detailed in Supplementary File), as previously described [35,36]. For patient use, this protocol was appropriately adopted as outlined in the Supplementary File. Radioiodination of [Tyr^4^]BBN with I-125 was accomplished following a known procedure [35].

Handling of solutions containing beta-/gamma-emitting radionuclides was conducted by authorized personnel in compliance with European radiation safety guidelines. Licensed facilities were supervised by the Greek Atomic Energy Commission (GAEC, license #A/435/17092/2019).

### 2.3. Cell Culture 

The human prostate adenocarcinoma PC-3 [38] and the ductal breast carcinoma T-47D cell-lines [39,40], both expressing the GRPR, were purchased from LGC Standards GmbH (Wesel, Germany). Cells were grown in Roswell Park Memorial Institute-1640 (RPMI-1640) medium with GlutaMAX-I, supplemented with 10% (*v*/*v*) fetal bovine serum (FBS), 100 U/mL penicillin, and 100 µg/mL streptomycin. Cells were kept in a controlled humidified air containing 5% CO_2_ at 37 °C and passages were carried out at 70–85% confluency using a trypsin/EDTA (0.05%/0.02% *w*/*v*) solution [36,41].

### 2.4. Competition Binding Assays in PC-3 Cell-Membranes 

The binding affinities of DB15 and [Tyr^4^]BBN (reference) were determined by displacement of the [^125^I]I-[Tyr^4^]BBN radioligand from freshly harvested PC-3 cell membranes, as previously described [35]. In brief, increasing concentrations of DB15 or [Tyr^4^]BBN (10^−13^–10^−6^ M) in triplicate were incubated with [^125^I]I-[Tyr^4^]BBN (30 pmol, ≈40,000 dpm) and membrane homogenate in binding buffer (300 µL, pH 7.4, 50 mM HEPES, 1% BSA, 5.5 mM MgCl_2_, 35 µM bacitracin) for 1 h at 22 °C. Rapid filtration through glass fiber filters (Whatman GF/B, presoaked in binding buffer for at least 1 h) on a Brandel Cell Harvester (Adi Hassel Ingenieur Büro, Munich, Germany) and rinsing with ice-cold washing buffer (10 mM HEPES pH 7.4, 150 mM NaCl) followed. Filter radioactivity was measured in a gamma counter (automated multi-sample well-type instrument with a NaI(Tl) 3” crystal, Canberra Packard Cobra^TM^ Quantum U5003/1, Auto-Gamma^®^ counting system). The 50% inhibitory concentration (IC_50_) was determined adopting nonlinear regression analysis according to a one-site model using PRISM 6 (Graph Pad Software, San Diego, CA, USA). Values are expressed as mean ± standard deviation (SD) of three experiments performed in triplicate.

### 2.5. Time-Dependent Uptake of [^99m^Tc]Tc-DB15 in PC-3 and T-47D Cells 

One day before the experiment, PC-3 or T-47D cells were seeded in 6-well plates (≈1 × 10^6^ cells per well). Next day, the cells were rinsed with ice-cold internalization medium (IM, RPMI-1640 GlutaMAX-I, supplemented by 1% (*v*/*v*) FBS). After adding fresh IM at 37 °C (1.2 mL), a further portion of IM (150 µL) was added in the upper well-row and [Tyr^4^]BBN solution in IM (150 µL) was added in the lower row (non-specific series). [^99m^Tc]Tc-DB15 (250 fmol total peptide in 150 µL 0.5% BSA-PBS) was added in all wells and the plates were incubated at for 15 min, 30 min, 1 h and 2 h at 37 °C in an Incubator-Orbital Shaker unit (MPM Instr. SrI, Bernareggio, MI, Italy). Cells were then placed on ice, the medium was collected, and the plates were rinsed with 0.5% BSA-PBS (1 mL). Membrane-bound fractions were collected after treatment in acid-wash solution (2 × 600 µL; 50 mM glycine buffer pH 2.8, 0.1 M NaCl). Internalized fractions were collected after treatment with 1 N NaOH (2 × 600 µL), as previously described [20,41]. After counting of radioactivity of all collected fractions in the gamma counter, the percentage of specific internalized and membrane-bound fractions per time point were calculated with Microsoft Excel and the respective curves were drawn. Specific internalized and membrane-bound counts were determined by subtracting the respective non-specific from the respective total counts. Results represent specific internalized ±SD of total added radioactivity per well from three experiments performed in triplicate.

### 2.6. In Vivo Metabolic Stability of [^99m^Tc]Tc-DB15 in Mice 

Each of three healthy male Swiss albino mice (30 ± 5 g, NCSR “Demokritos” Animal House Facility) received a bolus of [^99m^Tc]Tc-DB15 (100 µL, 55.5–111 MBq, 3 nmol of total peptide in vehicle: saline/EtOH 9/1 *v*/*v*) injected in the tail vein together with vehicle (100 µL; controls). A parallel group of mice received the same bolus injected together with PA (100 µL of vehicle containing 300 µg PA; PA group). Animals were euthanized 5 min post-injection (pi) and blood (0.5–1 mL) was collected from the heart using a pre-chilled syringe, swiftly placed in pre-chilled Eppendorf Protein LoBind Tubes (Eppendorf, Wesseling-Berzdorf, Germany) containing EDTA. Samples were processed as previously described [26,36] and were then analyzed by radioanalytical HPLC (for HPLC equipment description see Supplementary File). A Symmetry-Shield RP18 (5 µm, 3.9 mm × 20 mm) column (Waters, Germany) was eluted at a 1 mL/min flow rate with the following gradient system: 100% A/0% B to 50% A/50% B in 50 min; A = 0.1% TFA in H_2_O and B = MeCN.

### 2.7. Biodistribution of [^99m^Tc]Tc-DB15 in PC-3 and in T-47D Xenograft-Bearing Mice 

Biodistribution experiments were conducted in two sets of female severe combined immunodeficiency (SCID) mice 6-8 weeks of age, provided by the NCSR “Demokritos” Animal House Facility. In the first set (15.9 ± 1.8 g body weight, BW), animals were inoculated in their flanks with a freshly harvested sterile cell suspension of PC-3 cells (1.3 × 10^7^ cells) in 150 µL physiological saline, as previously described [35]. Animals were kept under aseptic conditions for 3.5 weeks until tumors of adequate size (140 ± 50 mg) were developed at the inoculation site and biodistribution was conducted (*vide infra*). In the second animal set (16.3 ± 1.6 g BW) mice were treated with estrogens in their drinking water (4 mg/L of ß-estradiol, Sigma-Aldrich, St. Louis, MO, USA) while being kept in an aseptic environment for a period of one week. Next, inocula of freshly harvested T-47D cells in Matrigel (Corning Life Sciences, Inc., Bedford, MA, USA) were prepared (150 µL suspension of 1.2 × 10^7^ cells) and subcutaneously (sc) injected in the flanks of mice. The animals were kept under estrogen treatment and aseptic conditions for further 9 weeks [11,41]. Within this period, they developed well-grown tumors (90 ± 30 mg) at the inoculation site and biodistribution was conducted as described below (the same procedure was followed for both sets of animals).

On the day of the experiment, mice in groups of four were injected through their tail vein with a [^99m^Tc]Tc-DB15 bolus (100 µL, 180-230 kBq, 10 pmol total peptide, in vehicle: saline/EtOH 9/1 *v*/*v*) together with either vehicle (100 µL; control groups—1, 4, and 24 h pi), or PA (300 µg PA dissolved in 100 µL vehicle; PA group—4 h pi), or excess [Tyr^4^]BBN (40 nmol [Tyr^4^]BBN dissolved in 100 µL vehicle; block group—4 h pi). At the selected time points animals were euthanized, dissected and blood, tumors and organs of interest were immediately collected, weighed and counted for their radioactivity content in the gamma counter along with appropriate standard solutions of the administered dose. Results were calculated as percent of administered dose per gram tissue (%IA/g) applying the Microsoft Excel program. Results were expressed as average %IA/g-values ± SD per time point.

All experiments involving mice were conducted in compliance with European and national regulations in licensed facilities (EL 25 BIO exp021). Applied protocols were approved by the Department of Agriculture and Veterinary Service of the Prefecture of Athens (protocol numbers #1609 for the stability and #1610 for the biodistribution studies).

### 2.8. Statistical Analyses 

Statistical evaluation of results was accomplished adopting a two-way ANOVA with multiple comparisons applying Tukey’s post hoc analysis (GraphPad Prism Software, San Diego, CA, USA). *p*-values of <0.05 were considered to be statistically significant.

### 2.9. Patient Studies 

Two BC patients were tested for the potential of pathological lesion detection after injection of [^99m^Tc]Tc-DB15. The study protocol was approved by the Bioethical Committee of the Poznan University of Medical Sciences (decision no. 1153 issued on 16 January, 2020) and patients gave their informed consent for their participation in the study. Both patients had histologically confirmed advanced disease, with several lesions identified by conventional imaging modalities (MRI, [^18^F]FDG PET/CT, mammography or breast USG), as detailed in the Supplementary File.

A single dose of [^99m^Tc]Tc-DB15 (5-mL, 700–800 MBq corresponding to 20 μg DB15) was administered as a slow intravenous (iv) injection over 1 min. Vital signs (pulse rate, systolic and diastolic BP) were measured prior to the injection of [^99m^Tc]DB15 as well as within 15 min pi. Likewise, ECGs were performed prior to the injection, continuously during the injection and for 15 min pi. ECG and vital signs were measured again after the last scan was completed and blood tests for hematology and biochemistry (glucose, creatinine, bilirubin, transaminases, sodium, potassium, calcium) were performed. The laboratory evaluation was repeated 7, 14 and 28 days after imaging. Adverse events (AEs) were assessed and graded according to National Cancer Institute Common Toxicity Criteria for Adverse Events (CTCAE) Version 3.0. 

Gamma camera imaging was performed on a Symbia Intevo Bold dual-head SPECT/CT scanner (Siemens Healthineers AG; Erlangen, Germany) equipped with a 16-slice CT scanner. Planar imaging of the whole body and SPECT/CT images of the thorax were obtained 15 min, 1 h, 3 h, and 24 h pi. Planar and SPECT/CT images were inspected for increased uptake by two experienced nuclear medicine specialists. 

## 3. Results

### 3.1. Radiolabeling—Quality Control 

Radiolabeling of DB15 with Tc-99m for preclinical testing (molecular activity of 20-30 MBq [^99m^Tc]Tc/nmol peptide) was accomplished by 30 min incubation of the radiolabeling reaction mixture, containing DB15, [^99m^Tc]TcO_4_^−^, SnCl_2_ and citrate anions, at pH 11 and at room temperature. For clinical testing, the labeling reaction was modified to reach a higher molecular activity (greater than 50 MBq [^99m^Tc]Tc/nmol peptide). 

In both cases, quality control of the radiolabeled product involved ITLC and HPLC methods (Supplementary File). Almost quantitative radiochemical yields (>98%) were established with less than 2% of total radiochemical impurities ([^99m^Tc]TcO_4_^−^, [^99m^Tc]Tc-citrate and [^99m^Tc]TcO_2_ × nH_2_O) present, whereas HPLC revealed the formation of a single radiochemical species (Figures S1 and S2; Supplementary File). Therefore, [^99m^Tc]Tc-D15 was used without further radiochemical purification [35]. 

### 3.2. Binding Affinity of DB15 for the Human GRPR 

**Figure 2:**
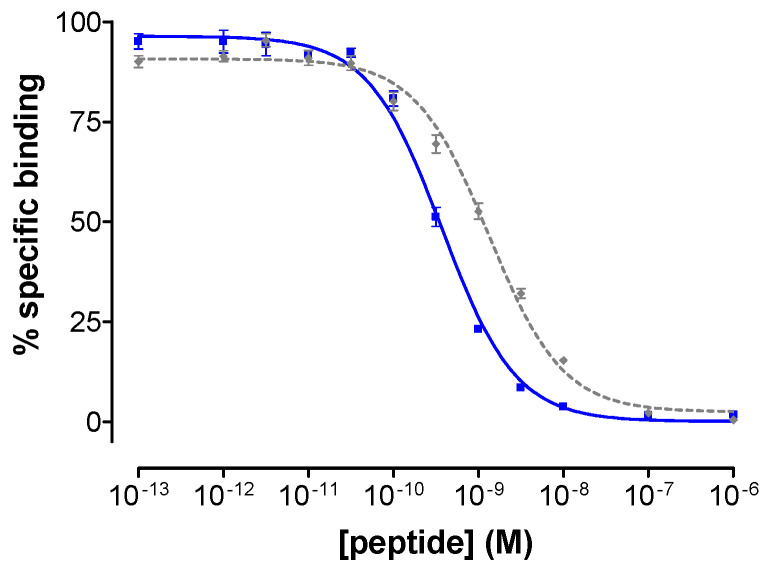
Displacement of [^125^I]I-[Tyr^4^]BBN by increasing concentrations of DB15 (■, IC_50_ = 0.37 ± 0.03 nM) or [Tyr^4^]BBN (◆ reference, 1.33 ± 0.09 nM) from PC-3 cell membrane homogenates; results represent average values ± SD of three experiments performed in triplicate.

As shown in Figure 2, DB15 displaced the [^125^I]I-[Tyr^4^]BBN radioligand from PC-3 cell membranes in a monophasic and dose-dependent way, displaying a higher binding affinity for the human GRPR (IC_50_ = 0.37 ± 0.03 nM, *n* = 3) compared with the [Tyr^4^]BBN reference (IC_50_ = 1.33 ± 0.09 nM, *n* = 3). 

### 3.3. GRPR-Specific Uptake of [^99m^Tc]Tc- DB15 by PC-3 and T-47D Cells 

**Figure 3:**
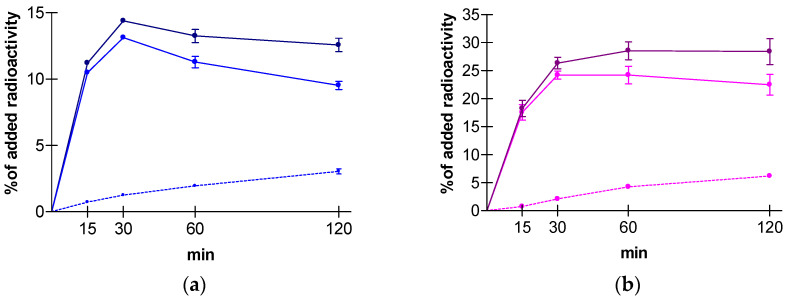
Time-dependent cell uptake curves of [^99m^Tc]Tc-DB15 in (**a**) PC-3 and (**b**) T-47D cells, including the 15 min, 30 min, 1 h, and 2 h intervals; cell-associated fractions (dark solid lines) comprise membrane-bound (light solid lines) plus internalized fractions (light dotted lines); results represent mean specific values ± SD of total added activity for each time point and are retrieved from three independent experiments performed in triplicate.

Time-dependent cell association curves of [^99m^Tc]Tc-DB15 in PC-3 and T-47D cells are shown in Figure 3. High and specific uptake was observed during 30 min incubation of [^99m^Tc]Tc-DB15 in PC-3 (13.1 ± 0.1%) and T-47D cells (24.2 ± 0.7%) at 37 °C. The majority of radioactivity was associated with the cell-membrane with a smaller portion (<6%) found within the cells, as expected for a GRPR-radioantagonist [20,35,41]. Cell association was banned (<0.9% at all time points, results not shown) in the presence of 1 μM [Tyr^4^]BBN, consistent with a GRPR-mediated process. 

### 3.4. Metabolic Stability of [^99m^Tc]Tc-DB15 in Mice 

**Figure 4:**
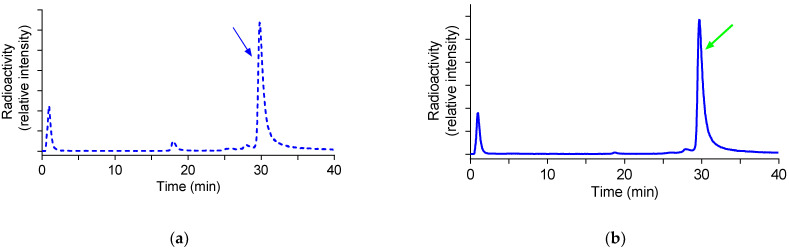
Representative gamma trace during HPLC analysis of blood collected 5 min pi of [^99m^Tc]Tc-DB15 in healthy mice with (**a**) vehicle co-injection (dotted line; 77.0% intact), or (**b**) with PA co-injection (solid line; 82.5% intact); the *t*_R_ of the radioligand is indicated by the arrows.

The stability of [^99m^Tc]Tc-DB15 in peripheral mouse blood was studied by HPLC analysis of blood samples collected 5 min pi in two animal groups, in untreated controls or in mice treated with the NEP-inhibitor PA. As shown by the representative radiochromatograms of Figure 4, [^99m^Tc]Tc-DB15 remained 76.4 ± 2.3% intact in controls (*n* = 3) with a minor but not statistically significant increase in the PA group (83.0 ± 2.3% intact, *n* = 3; *p* > 0.05). 

### 3.5. Biodistribution of [^99m^Tc]Tc-DB15 in PC-3 and in T-47D Xenograft-Bearing Mice

**Figure 5:**
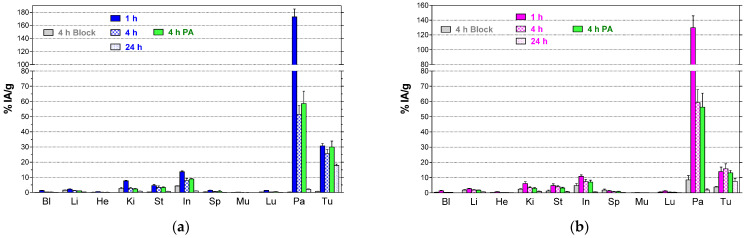
Biodistribution of [^99m^Tc]Tc-DB15 in (**a**) PC-3 and (**b**) T-47D tumor bearing mice at 1, 4, and 24 h pi (%IA/g, mean ± SD, *n*= 4); two separate 4-h interval groups of animals were included representing mice co-injected either with excess [Tyr^4^]BBN (block), or with the NEP-inhibitor PA (PA); Bl = blood, Li = liver, He = heart, Ki = kidneys, St = stomach, In = intestines, Sp = spleen, Mu = muscle, Lu = lungs, Pa = pancreas and Tu = tumor (PC-3 in (**a**) and T-47 D in (**b**)).

Biodistribution results of [^99m^Tc]Tc-DB15 in SCID mice bearing PC-3 or T-47D xenografts at 1, 4 and 24 h pi are shown in Figure 5, as %IA/g±SD, *n* = 4. Results in numerical values are summarized in Tables S1 and S2, respectively, in the Supplementary File. The radioligand cleared fast from blood and background tissues via the kidneys into urine. [^99m^Tc]Tc-DB15 rapidly and specifically localized in the PC-3 (30.70 ± 2.76 %IA/g at 1 h pi) and T-47D (14.01 ± 2.87 %IA/g at 1 h pi) tumors showing good retention (PC-3: 17.79 ± 1.58 %IA/g / T-47D: 7.55 ± 1.81 %IA/g at 24 h pi). High uptake was also observed in the GRPR-expressing mouse pancreas (>130 %IA/g at 1 h pi), which however rapidly declined with time (~2 %IA/g at 24 h pi), as consistent with a radioantagonist profile [20,35,41]. Treatment of animals with PA did not induce any significant change in the tumor uptake, either for the PC-3 (controls: 25.56 ± 2.78 %IA/g vs PA-treated: 30.03 ± 3.90 %IA/g at 4 h pi; *p* > 0.05) or the T-47D implants (controls: 15.82 ± 3.20 %IA/g vs. PA-treated: 13.15 ± 1.55 %IA/g at 4 h pi; *p* > 0.05), in agreement with the minor impact of PA on the in vivo stability of [^99m^Tc]Tc-DB15 in peripheral mice blood. In all cases radioligand uptake was receptor-mediated, as suggested by the significant reduction (*p* < 0.001) of tumor and pancreas values during in vivo GRPR-blockade by co-injection with excess [Tyr^4^]BBN. For example, the uptake in the PC-3 tumors was drastically reduced from 25.56 ± 2.78 %IA/g in controls to 0.72 ± 0.12 %IA/g in the block-group of mice at 4 h pi (*p* < 0.0001).

### 3.6. First Results of [^99m^Tc]Tc-DB15 in BC Patients Applying SPECT/CT

#### 3.6.1. Tolerability

No adverse reactions were recorded after injection of [^99m^Tc]Tc-DB15. The patients did not report any specific signs, the heart rate and blood pressure were stable during and after injection. No significant changes in the hematology and biochemistry parameters were recorded at any time point after the imaging. Patient 1 complained of intensified bone pain (CTCAE grade 1) several hours after the scan, which however resolved after a few days and was attributed to the forced supine position during image acquisition.

#### 3.6.2. Physiological Tissue Distribution

**Figure 6:**
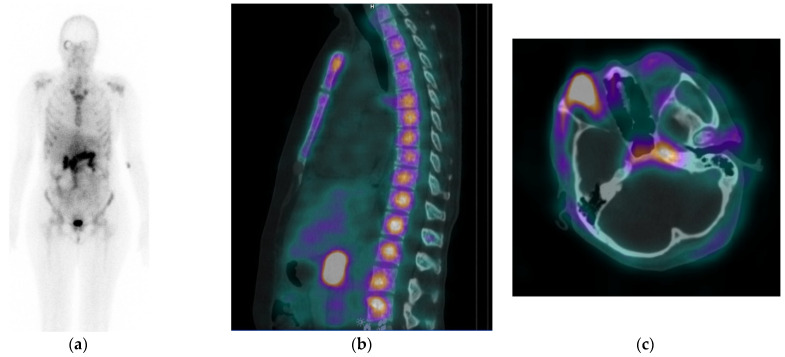
(**a**) Whole body scan of Patient 1, obtained 3 h after injection of [^99m^Tc]Tc-DB15 in the anterior projection showing physiological accumulation in the pancreas and increased uptake in the skeleton. (**b**) SPECT/CT sagittal image obtained 3.5 h after injection of [^99m^Tc]Tc-DB15 showing increased accumulation in the vertebrae. (**c**) SPECT/CT transaxial image showing elevated accumulation in the right orbital wall and in the sphenoid bone.

**Figure 7:**
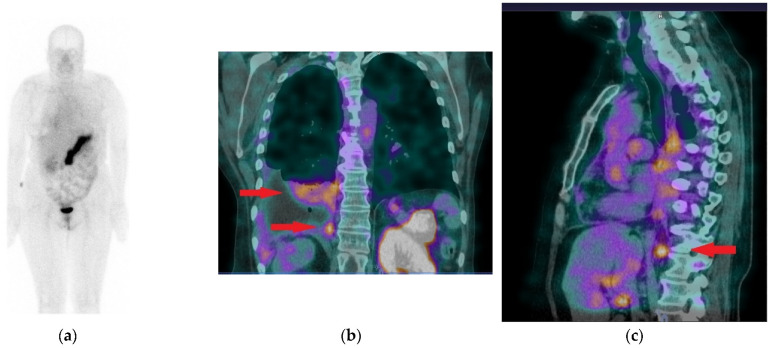
(**a**) Whole body scan of Patient 2, obtained 3 h after injection of [^99m^Tc]Tc-DB15 in the anterior projection shows physiological accumulation in the pancreas and increased uptake in the right pleura. (**b**) SPECT/CT coronal image of the chest presenting increased tracer uptake in the metastatic infiltrations in the pleura and lung parenchyma (red arrows). (**c**) SPECT/CT sagittal image of the chest depicting increased radioactivity accumulation in an enlarged (metastatic) phrenic lymph node (red arrow).

Planar and SPECT/CT images showed accumulation of [^99m^Tc]Tc-DB15 in the pancreas of both patients (Figure 6 and Figure 7). The bulk of the radioactivity rapidly cleared via the kidneys into urine in the first 2 h, with a smaller portion being slowly eliminated via the liver and visible in the bowels at 24 h pi. 

#### 3.6.3. Pathological Findings

The planar and SPECT/CT images revealed increased uptake of [^99m^Tc]Tc-DB15 in the BC metastases in both patients. Patient history and further information concerning the study can be found in the Supplementary File. Patient 1 displayed increased [^99m^Tc]Tc-DB15 uptake in the bones (spine, sternum, ribs and pelvis), which correlated with osteosclerotic lesions on the CT scan (Figure 1 and Figure S3 in Supplementary File) as well as with bone lesions revealed by [^18^F]FDG PET/CT (Figure S4; Supplementary File). However, [^99m^Tc]Tc-DB15 failed to demonstrate intraperitoneal metastases during SPECT/CT which were identified by [^18^F]FDG PET/CT and subsequently confirmed in histopathology (not including GRPR-expression status). 

In patient 2, increased tracer accumulation was visible in the thickened pleura of the right lung corresponding to pleural BC metastases. Additionally, increased uptake was noted in an enlarged right phrenic lymph node (Figure 7). 

## 4. Discussion

As a part of our ongoing work on anti-GRPR radioligands for application in cancer theranostics [20,29,34,35,36], we now present [^99m^Tc]Tc-DB15, a radiotracer based on the potent GRPR-antagonist [_D_Phe^6^,LeuNHEt^13^]BBN(6-13) [30,31] and, thus, associated with better safety for human use. The acyclic tetraamine framework positioned at the N-terminus allowed labeling with Tc-99m, a broadly available radiometal ideally suited for high-quality SPECT and SPECT/CT imaging [33]. Metabolic stability of peptide radioligands in the blood stream represents an important prerequisite for good tumor targeting by ensuring the sufficient delivery of intact molecules to tumor lesions expressing the target. Previous studies have shown that the fast in vivo degradation of BBN-like peptides is driven by neutral endopeptidase (NEP) [42,43]. Most importantly, it was demonstrated that in-situ inhibition of NEP induced by administration of appropriate inhibitors, such as phosphoramidon (PA) or Entresto^®^, could provoke significant increases in the tumor uptake of several anti-GRPR radiopeptides through their stabilization in peripheral blood [25,26,36,44,45]. 

Interestingly, four NEP-cleavage sites could be identified in related [_D_Phe^6^,LeuNHEt^13^]BBN(6-13)-based radiopeptides, namely, the His^12^-Leu^13^, Ala^9^-Val^10^, Trp^8^-Ala^9^, and Gln^7^-Trp^8^ bonds [26]. Although neither the Val^10^-Gly^11^ nor the Gly^11^-His^12^ peptide bond were hydrolyzed by NEP, still the position 11 residue turned out to be critical for modulating resistance to the enzyme. For example, replacement of Gly^11^ by DAla^11^ led to more metabolically robust radioligands (about 75% intact DAla^11^-modified radiopeptides vs. 55–60% intact molecules detected in the respective Gly^11^-original analogs at 5 min pi in mice). However, such increases failed to eventually translate into better tumor uptake, because other critical parameters (e.g., cell uptake capabilities, or pharmacokinetics) were compromised [35,36,37]. A comparable metabolic stability was achieved by our Sar^11^-tracer, [^99m^Tc]Tc-DB15 (76.4 ± 2.3% intact radiotracer in peripheral mouse blood at 5 min pi), confirming once more the significance of position 11 residue on stability. Interestingly, treatment of mice with PA failed to induce significant increases of stability (83.0 ± 2.3% intact, *n* = 3; *p* > 0.05), thereby virtually revealing full resistance of [^99m^Tc]Tc-DB15 to NEP. Yet unlike the DAla^11^ analogs, [^99m^Tc]Tc-DB15 preserved high GRPR-specific cell binding capabilities in both PC-3 and T-47D cells.

It is interesting to observe how the above promising qualities of [^99m^Tc]Tc-DB15 translated in biodistribution patterns in mice bearing GRPR-positive tumors. Firstly, the radiotracer displayed a high and GRPR-specific uptake in both the PC-3 and the T-47D xenografts at all time points. Secondly, the high %IA/g values at 24 h pi reveal the advantageous retention of [^99m^Tc]Tc-DB15 in the experimental tumors. Thirdly, background radioactivity declined rapidly, especially from the GRPR-rich mouse pancreas. As a result of the above, [^99m^Tc]Tc-DB15 displayed a quite attractive in vivo profile with tumor-to-background ratios increasing with time. Thus, for example, the uptake of [^99m^Tc]Tc-DB15 in the PC-3 xenografts remained as high as 17.79 ± 1.58 %IA/g even at 24 h pi with the pancreatic uptake conversely declining to 2.07 ± 0.62 %IA/g, illustrating the excellent biodistribution pattern of the Sar^11^-radiotracer. It should be noted that the respective values for the non-modified Gly^11^-analog were previously reported to be 16.32 ± 1.82 %IA/g for the PC-3 tumors and 30.26 ± 14.65 %IA/g for the pancreas [35]. Prolonged retention in the tumor is an attractive quality for a theranostic GRPR-seeking radiolabeled probe, agonist, or antagonist, especially during radionuclide therapy. This fact has been illustrated in a recent report, whereby cysteine cathepsin inhibitors are coupled to GRPR-peptides leading to improved tumor retention via endolysosomal trapping [46]. Another interesting finding of the current biodistribution study has been the lack of improvements in the tumor uptake in the mice treated with PA vs. the untreated controls at 4 h pi. Indeed, no significant difference was observed in either the PC-3 or the T-47D xenografts during in-situ NEP-inhibition, concordant with findings from the in vivo stability study, which ruled out the involvement of NEP in the degradation of circulating [^99m^Tc]Tc-DB15.

The above promising preclinical properties of [^99m^Tc]Tc-DB15 prompted us to explore its clinical applicability in the detection of GRPR-positive lesions in BC and PC patients. Previous studies with [^68^Ga]Ga-labeled GRPR-antagonist SB3 (SB3, [DOTA-*p*-AMA-DGA-_D_Phe^6^,LeuNHEt^13^]BBN(6-13)) revealed the safety and feasibility of detecting GRPR-expressing pathological lesions of advanced BC and PC patients applying [^68^Ga]Ga-SB3 and PET/CT [29] with a more recent study in therapy-naïve PC patients revealing better results and reporting excellent correlation of imaging findings with GRPR-expression levels in the primary PC excised lesions [7]. 

Our first experience with [^99m^Tc]Tc-DB15 and SPECT/CT was acquired in two BC patients with disseminated disease. Both patients tolerated the [^99m^Tc]Tc-DB15 injection, showing no adverse effects thereafter and during follow up. During imaging, the bone metastases revealed by [^99m^Tc]Tc-DB15 in patient 1 correlated well with those detected by [^18^F]FDG PET/CT and CT. However, disease infiltrated to peritoneum taking up [^18^F]FDG on PET/CT was not visible on [^99m^Tc]Tc-DB15 SPECT/CT imaging. It should be noted however that GRPR-expression levels were not determined in the samples acquired by laparotomy for histological confirmation of BC. In the second patient with advanced BC infiltrating in the pleura, as confirmed by histopathology, high uptake of [^99m^Tc]Tc-DB15 was shown on SPECT/CT in the lower lobe of the lung and additionally in an enlarged phrenic lymph node. The latter could not be confirmed histologically as a BC metastasis because of anatomical position restraining surgical intervention. Again, the GRPR-expression status was not determined in the samples taken from this patient either. The above preliminary clinical results are encouraging in terms of biosafety. They also seem rather positive with regards to efficacy, especially when the high heterogeneity of primary and metastatic BC, including GRPR-expression levels, is taken into account [9,10]. Yet, many open questions have to be rigorously addressed before confirming the diagnostic value of [^99m^Tc]Tc-DB15 in BC and potentially in other human cancers too. Firstly, we need to correlate imaging findings with histologically established data on GRPR-expression in a systematic way. Then, we need to understand if and to what extent additional parameters, such as BC type and stage along with preceding therapies, affect GRPR-expression levels on the lesions and thereby diagnostic accuracy. Hence, further clinical evaluation of [^99m^Tc]Tc-DB15 appears to be warranted.

## 5. Conclusions

We have introduced [^99m^Tc]Tc-DB15, a GRPR-antagonist based radiotracer, as a candidate for diagnostic imaging of GRPR-positive human tumors. In addition to the inherent biosafety of an antagonist, labeling with the preeminent nuclear medicine radionuclide Tc-99m allows for excellent quality images using broadly available SPECT and SPECT/CT instrumentation. Substitution of Gly^11^ by Sar^11^ in the peptide backbone, has led to high metabolic resistance to NEP, a major catabolizing protease of BBN-like peptides in vivo. Unlike previously attempted DAla^11^/Gly^11^ substitutions, [^99m^Tc]Tc-DB15 retained high cell binding efficacy in both prostate PC-3 and in BC T-47D cells in vitro. Most interestingly, it displayed high uptake and prolonged retention in the respective PC-3 and T-47D xenografts grown in mice. These qualities combined with a rapid background clearance, resulted in an excellent pharmacokinetic profile. Translation in two advanced BC patients, resulted in no side effects, confirming previous observations on the biosafety of radiotracers based on the potent GRPR-antagonist [DPhe^6^,LeuNHEt^13^]BBN(6-13) and on GRPR-antagonist radioligands in general. Furthermore, it revealed the ability of [^99m^Tc]Tc-DB15 to detect several metastatic BC lesions, both in the skeleton and in soft tissues, but these findings need to be confirmed prospectively in a dedicated human study. In view of the above, further clinical evaluation seems to be warranted to establish the diagnostic value of [^99m^Tc]Tc-DB15 in BC, PC, and other GRPR-expressing human malignancies.

## Data Availability

The data presented in this study are available in this article (and supplementary material).

## Supplementary Materials

The following are available online at https://www.mdpi.com/article/10.3390/cancers13205093/s1, Figure S1: Typical radiochromatogram of HPLC analysis of [^99m^Tc]Tc-DB15 (preclinical); Figure S2: Typical radiochromatogram of HPLC analysis of [^99m^Tc]Tc-DB15 (for patients); Figure S3: Whole body scan 3 h pi of [^99m^Tc]Tc-DB15 in patient 1 (with anterior and posterior projection); Figure S4: PET/CT 1 h pi of [^18^F]FDG in patient 1; Table S1: Numerical biodistribution data for [^99m^Tc]Tc-DB15 in PC-3 xenograft-bearing SCID mice at 1, 4 and 24 h pi; Table S2: Numerical biodistribution data for [^99m^Tc]Tc-DB15 in T-47D xenograft-bearing SCID mice at 1, 4 and 24 h pi.
