# Supplementary material for: [99mTc]Tc-DB15 in GRPR-Targeted Tumor Imaging with SPECT: From Preclinical Evaluation to the First Clinical Outcomes
Source: Cancers (Basel). 2021 Oct 12;13(20):5093. doi: 10.3390/cancers13205093 (PMC8533986; doi:10.3390/cancers13205093)
Supplement: Supplementary file 1 [file cancers-13-05093-s001.zip › cancers-1402961-supplementary.pdf]

SUPPLEMENTARY FILE

[^{99m}Tc]Tc-DB15 in GRPR-targeted tumor imaging with SPECT: From preclinical evaluation to the first clinical outcomes

Berthold A. Nock [1], Aikaterini Kaloudi [1], Panagiotis Kanellopoulos [1], Barbara Janota [2], Barbara Bromińska [3], Dariusz Iżycki [4], Renata Mikołajczak [2], Rafał Czepczynski [3], and Theodosia Maina [1,*]

[1]Molecular Radiopharmacy, INRaSTES, NCSR "Demokritos", 15310 Athens, Greece
[2]National Centre for Nuclear Research, Radioisotope Centre POLATOM, 05-400 Otwock -Świerk, Poland
[3]Department of Endocrinology, Metabolism and Internal Diseases, Poznan University of Medical Sciences, 60-355 Poznań, Poland
[4]Department of Cancer Immunology, Poznan University of Medical Sciences, 61-866 Poznań, Poland

*Correspondence: maina_thea@hotmail.com; Tel.: +30-650-3908/3891

HPLC system for radioanalytical control of preclinically applied [^{99m}Tc]Tc-DB15.

For the HPLC analysis of [^{99m}Tc]Tc-DB15 prepared for preclinical application, a Waters chromatography system was employed. Twin detection modes were applied comprising a 2998 photodiode array UV detector (UV trace; Waters, Vienna, Austria) and a Gabi gamma detector (gamma trace; Raytest RSM Analytische Instrumente GmbH, Straubenhardt, Germany). The system was controlled by the Empower Software (Waters, Milford, MA, USA). For radioanalytical testing, a Symmetry Shield RP-18 (5 μm, 3.9 mm × 150 mm) cartridge column (Waters, Eschborn, Germany) was eluted at a 1 mL/min flow rate with a linear gradient: starting from 100%A/0% B and advancing to 60%A/40% B within 30 min (solvent A = 0.1% aqueous TFA and B = MeCN). A representative radiochromatogram is included below in Figure S1.

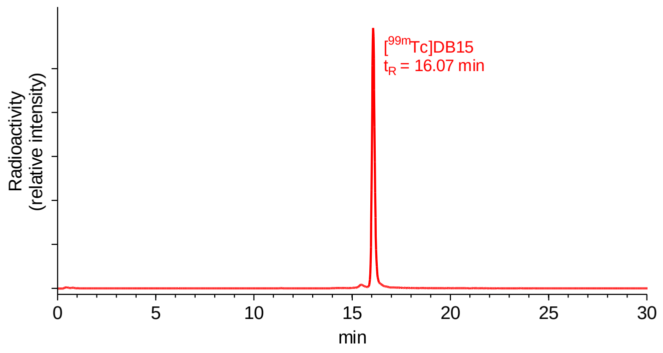

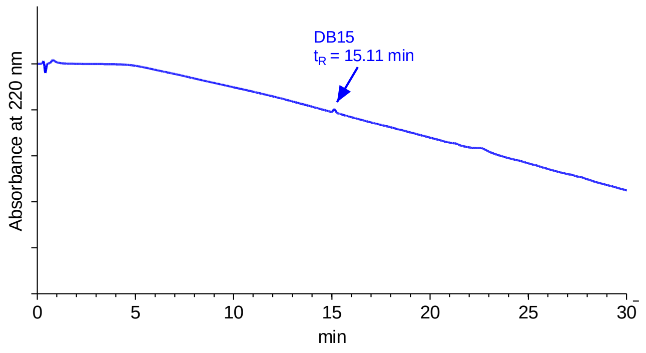

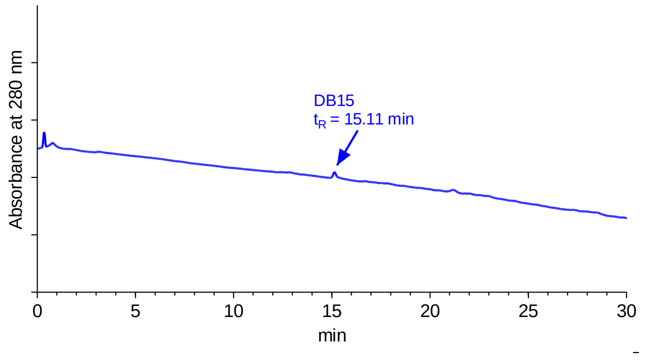

Figure S1. Typical radiochromatogram of HPLC analysis of [^{99m}Tc]Tc-DB15 labeling reaction mixture, showing the quantitative formation of a high purity radioligand on the upper part (t_R = 18.13 min, yield 99.7%, purity 100%); in the middle and lower diagrams the photometric (UV) trace of the labeling reaction mixture is also included at 220 and 280 nm, respectively, showing the non-labeled compound DB15 eluting as a single species at t_R = 25.11 min.

Preparation of [99mTc]Tc-DB15 for patient application.

DB15 in lyophilized form was dissolved in H_2O to a final concentration of 1 mM. The bulk solution of DB15 was distributed in 50-µL aliquots in Eppendorf Protein LoBind Tubes (Eppendorf, Wesseling-Berzdorf, Germany) which were stored at -20^{0}C. A labeling reaction mixture of 0.5 M phosphate buffer (0.5 mol/L, 25 µL; pH 11.0), 0.1 M sodium citrate solution (0.1 mol/L, 5 µL), DB15 solution (14 nmol, 14 µL), [^{99m}Tc]$NaTcO_4$ generator eluate (740 MBq, 500 µL; a commercial [^{99}Mo]Mo/[^{99m}Tc]Tc generator; Poltechnet 8.0 – 175 GBq, POLATOM, Poland) and a freshly prepared $SnCl_2$ solution in EtOH (4 mmol/L, 16 µL) was incubated at room temperature for 30 min. The mixture was then neutralized to pH 7-8 by adding NaH_2PO_4 (1 mol/L, 5µL) and sterile filtration was performed using a 0.22 µm Millex-GV filter (Millipore; Milford, MA, USA).

Quality control of [99mTc]Tc-DB15 used in patients

The quality control of [^{99m}Tc]Tc-DB15 prepared for patient use combined instant TLC (ITLC) and HPLC methods. ITLC was performed on silica gel strips (iTLC SG-glass microfiber chromatography plates impregnated with silica gel, 2 cm x 10 cm) developed with either 2-Butanone to assess the amount of free [^{99m}Tc]$TcO_4{}^-$ (R_f = 1), or 5 M aq. CH_3COONH_4/MeOH (1: 1, v: v) to reveal the formation of [^{99m}Tc]Tc-colloid (R_f = 0).
For HPLC analysis an Xterra RP-18 (5 µm, 3.9 mm x 20 mm) column (Waters, Vienna, Austria) was used on a Schimadzu chromatography system (Columbia, MD, USA). The column was eluted as follows: flow rate 1 mL/min, gradient elution: 0 min — 100% A/0% B, 30 min — 40% A/60% B, 35 min — 100% A/0% B, mobile phase A: 0.1% TFA in H_2O, B: 0.1% TFA in MeCN. A representative radiochromatogram is included below in Figure S2.

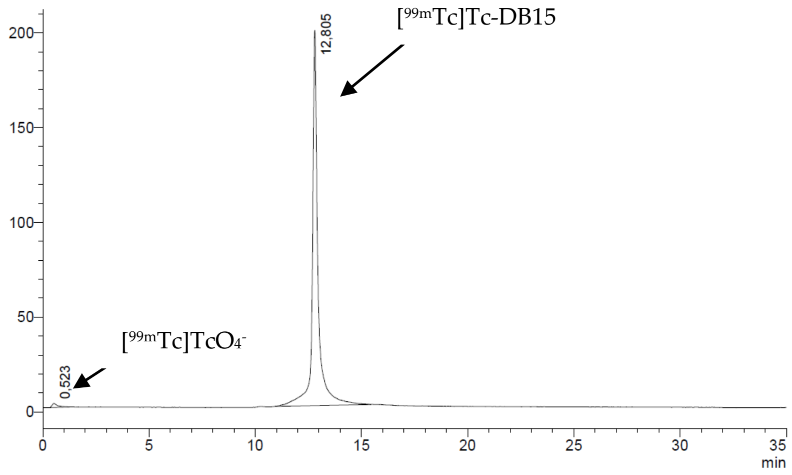

Figure S2. Representative radiochromatogram of HPLC analysis of [^{99m}Tc]Tc-DB15 prepared for patient administration, showing a high purity formation of the radioligand (t_R= 12.8 min, radiochemical purity 98.9%).

Biodistribution of [^{99m}Tc]Tc-DB15 in PC-3 or T-47D Xenograft-Bearing Mice

Table S1. Biodistribution data for [^{99m}Tc]Tc-DB15, expressed as %IA/g mean±sd, n=4, in PC-3 xenograft-bearing SCID mice at 1, 4 and 24 h pi.

Tissue	1 h	4 h – Block [1]	4 h	4 h – PA [2]	24 h
Blood	1.32 ± 0.16	0.08 ± 0.01	0.33 ± 0.09	0.28 ± 0.05	0.06 ± 0.02
Liver	2.19 ± 0.31	1.49 ± 0.18	1.20 ± 0.26	1.04 ± 0.09	0.31 ± 0.03
Heart	0.64 ± 0.04	0.14 ± 0.07	0.16 ± 0.04	0.17 ± 0.04	0.04 ± 0.01
Kidneys	7.63 ± 0.70	2.71 ± 0.56	2.80 ± 0.55	2.33 ± 0.37	0.86 ± 0.10
Stomach	4.58 ± 1.35	0.41 ± 0.01	3.32 ± 0.99	3.27 ± 0.42	0.74 ± 0.18
Intestines	13.64 ± 1.21	4.27 ± 0.19	7.83 ± 1.50	8.88 ± 0.34	0.98 ± 0.22
Spleen	1.44 ± 0.33	0.41 ± 0.08	0.56 ± 0.19	0.74 ± 0.63	0.14 ± 0.05
Muscle	0.23 ± 0.03	0.06 ± 0.05	0.05 ± 0.01	0.04 ± 0.01	0.02 ± 0.01
Lungs	1.42 ± 0.06	0.30 ± 0.12	0.33 ± 0.08	0.48 ± 0.19	0.11 ± 0.06
Pancreas	173.00 ± 24.06	0.26 ± 0.05	51.43 ± 5.74	58.58 ± 8.13	2.07 ± 0.62
Tumor	30.71 ± 2.76	0.72 ± 0.12	25.56 ± 2.78	30.03 ± 3.90	17.79 ± 1.58

All animals were injected with 180 – 230 kBq/10 pmol peptide; [1] animals co-injected with 50 μg [Tyr^4]BBN for in vivo GRPR-blockade; [2] animals co-injected with 300 μg PA for in situ inhibition of NEP.

Table S2. Biodistribution data for [^{99m}Tc]Tc-DB15, expressed as %IA/g mean±sd, n=4, in T-47D xenograft-bearing SCID mice at 1, 4 and 24 h pi.

Tissue	1 h	4 h – Block [1]	4 h	4 h – PA [2]	24 h
Blood	1.30 ± 0.34	0.29 ± 0.23	0.33 ± 0.04	0.29 ± 0.05	0.03 ± 0.01
Liver	2.67 ± 0.30	1.78 ± 0.29	1.89 ± 0.29	1.75 ± 0.15	0.58 ± 0.06
Heart	0.62 ± 0.15	0.20 ± 0.05	0.25 ± 0.01	0.19 ± 0.04	0.08 ± 0.02
Kidneys	6.06 ± 1.23	2.39 ± 0.35	3.38 ± 0.48	2.86 ± 0.54	0.86 ± 0.27
Stomach	4.76 ± 1.16	1.22 ± 0.58	4.01 ± 0.68	3.17 ± 0.51	0.71 ± 0.16
Intestines	10.82 ± 0.99	4.80 ± 1.28	7.32 ± 1.53	7.01 ± 1.33	0.55 ± 0.15
Spleen	1.27 ± 0.22	1.80 ± 0.63	0.75 ± 0.24	0.84 ± 0.18	0.18 ± 0.05
Muscle	0.19 ± 0.02	0.04 ± 0.01	0.06 ± 0.01	0.05 ± 0.01	0.01 ± 0.01
Lungs	1.14 ± 0.20	0.46 ± 0.18	0.45 ± 0.11	0.37 ± 0.07	0.07 ± 0.01
Pancreas	129.77 ± 16.11	8.51 ± 2.82	59.27 ± 8.47	56.23 ± 9.18	1.86 ± 0.71
Tumor	14.01 ± 2.87	3.82 ± 0.30	15.82 ± 3.20	13.15 ± 1.55	7.55 ± 1.81

All animals were injected with 180 – 230 kBq/10 pmol peptide; [1] animals co-injected with 50 μg [Tyr^4]BBN for in vivo GRPR-blockade; [2] animals co-injected with 300 μg PA for in situ inhibition of NEP.

SPECT/CT with [^{99m}Tc]Tc-DB15 in Breast Cancer Patients

Patient 1

A 63 year-old woman diagnosed with a bilateral metastatic breast cancer (invasive lobular carcinoma (ILC); ER-positive; PR-positive; HER2-negative, Ki-67-10%). Bone metastases were present already at the time of diagnosis. Due to the advanced stage, she was treated with hormonal therapy (letrozole). Subsequently and due to disease progression, the patient received the second line treatment with fulvestrant and palbocyclib. At the time of the study, the patient complained of a generalized bone pain attributed to bone metastases.

Planar and SPECT/CT findings were compared to other imaging methods: breast USG, mammography, [^{18}F]FDG PET/CT, magnetic resonance imaging (MRI).

In all the scans an increased [^{99m}Tc]Tc-DB15 uptake was seen in the bones (spine, sternum, ribs and pelvis), that correlated with osteosclerotic lesions on the CT scan. The bone metastases showed also increased uptake of [^{18}F]FDG (SUVmax of 4.4) in a PET/CT study performed 5 days later. In addition, the MRI confirmed multiple bone metastases in the skeleton.

A particularly high [^{99m}Tc]Tc-DB15 accumulation was noted in the superior wall of the right orbit (visible in the planar image Figure S3A and SPECT/CT – Figure S4C). The patient did not report any specific complaints related to the right eye or orbit. We did not find any correlation of this focus in the PET/CT and MRI images. In order to verify the finding, the patient underwent also ophthalmological examination and orbital ultrasonography, but no clear orbital pathology was diagnosed. The finding has been interpreted as a metastatic area in the bone with a higher expression of GRPR.

Furthermore, [^{18}F]FDG PET/CT revealed infiltration of peritoneum with a focal [^{18}F]FDG uptake (SUVmax 4.7). The patient was subjected to a diagnostic laparotomy that confirmed metastases of primary breast cancer to peritoneum (ER-positive; PR- positive; HER2-negative, Ki-67-10-15%; no GRPR-expression status was included). During direct comparison of [^{18}F]FDG PET/CT and [^{99m}Tc]Tc-DB15 SPECT/CT, no increased accumulation of [^{99m}Tc]Tc-DB15 was evident in the abdomen.

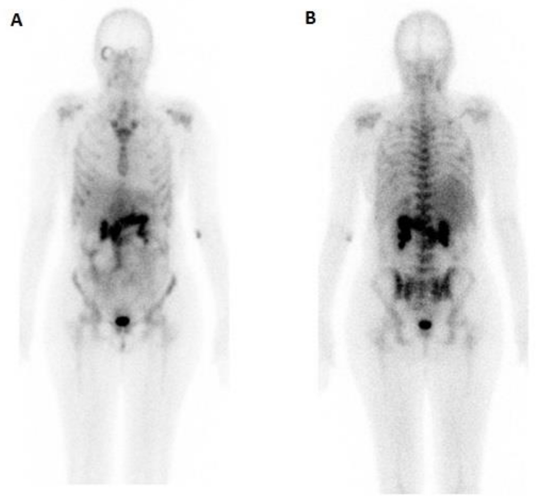

Figure S3. Whole body scan obtained 3 h after injection of [^{99m}Tc]Tc-DB15 in the anterior (A) and posterior (B) projection revealing physiological accumulation in the pancreas and increased pathological uptake in the skeleton.

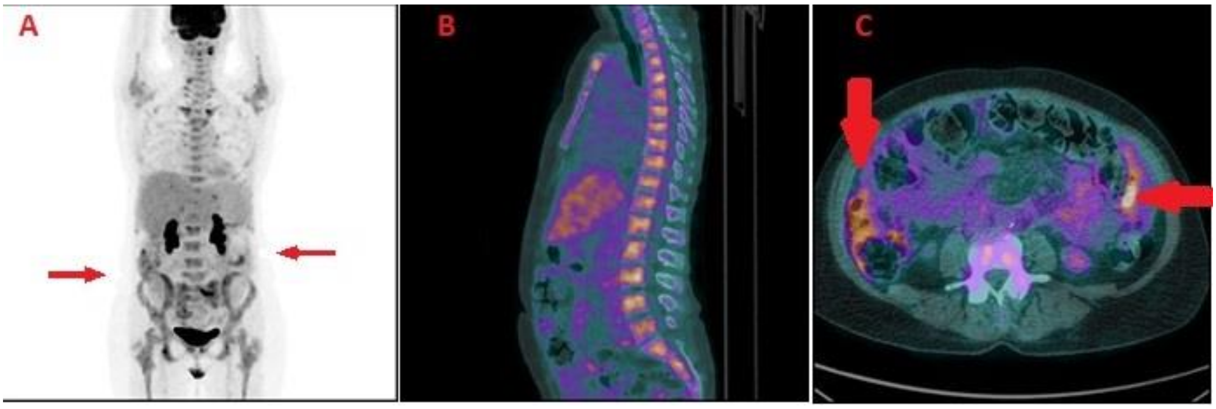

Figure S4. PET/CT showing diffuse uptake of [^{18}F]FDG in the skeleton (bone metastases) in the maximal intensity projection (A) and in the sagittal section of the spine (B). Increased [^{18}F]FDG uptake in the abdominal metastases (red arrows) was visible also in the transaxial image of the abdomen (C). PET/CT was performed 60 min after injection of [^{18}F]FDG (285 MBq, Synektik, Poland) on a Discovery IQ PET/CT scanner (GE Healthcare, Chicago/Il, USA).

Patient 2

Patient 2 was a 62 year-old woman with a diagnosis of pleural breast cancer metastases. The patient was treated with left mastectomy and chemotherapy at 44 years of age and complete remission was achieved. However, three months prior to the study the patient had been experiencing dyspnea, decreased exercise tolerance and loss of weight (5 kg in 3 months). A computed tomography (CT) of the chest revealed high effusion in the right pleura, irregular thickening and nodules in the right pleura. The fluid was evacuated and specimens of the pleura were obtained by means of video-assisted thoracoscopic surgery for histopathological examination, which confirmed pleural metastases of ductal breast carcinoma (ER-positive and PR-positive in 100% of the cells).

Increased accumulation of [^{99m}Tc]Tc-DB15 was visible in the thickened pleura of the lower lobe of the right lung. Additionally, increased uptake was noted in an enlarged right phrenic lymph node (dimensions: 16 mm x 9 mm) – Figure 6. Histological verification of this lymph node could not be performed due to the anatomical location.
